# Leveraging dissemination and implementation science to facilitate adoption of a human nutrition research e-learning course

**DOI:** 10.1017/cts.2025.44

**Published:** 2025-03-05

**Authors:** Denise Daudelin, Penny M. Kris-Etherton, Alyssa Cabrera, Anna L. Thompson, Kris M. Markman, Linfei Chen, Alice H. Lichtenstein

**Affiliations:** 1 Tufts Clinical and Translational Science Institute, USA; 2 Tufts University, USA; 3 Department of Nutritional Sciences, Penn State University, USA; 4 Jean Mayer USDA Human Nutrition Research Center on Aging, Tufts University, USA

**Keywords:** Dissemination and implementation science, consolidated framework for Implementation Research (CFIR), value-added Research Dissemination Framework, human nutrition research, workforce development

## Abstract

**Background:**

Tufts Clinical and Translational Science Institute (CTSI) developed an online self-paced course to address the gap identified in critical thinking skills related to peer-reviewed nutrition science publications. Initial engagement was low, prompting the launch of a quality improvement project utilizing Dissemination and Implementation (D&I) science principles to enhance participation. This report details the development and execution of the dissemination strategy, course promotion methods, and outcomes related to participant engagement and feedback.

**Methods:**

A dissemination plan was designed and implemented using the Value-Added Research Dissemination Framework and the Consolidated Framework for Implementation Research (CFIR). Dissemination efforts targeted registered dietitians and university nutrition program instructors, along with their students.

**Results:**

During the active dissemination period from January to May 2023, the cumulative numbers of learners increased from 23 to 118. Instructors from three nutrition degree programs found the course valuable, reporting that it introduced new content or reinforced existing material. Learner participation continued past the active dissemination period into 2024. Findings from the course evaluation survey provided insights to guide future course improvements.

**Conclusion:**

This project demonstrates the successful use of D&I frameworks to support the dissemination and implementation of educational innovations such as online learning initiatives.

## Introduction

An important barrier to translating research into clinical practice for healthcare professionals is a lack of training in the critical evaluation of research literature [1]. This skill gap is particularly important for nutrition and dietetics professionals, as well as primary care physicians, given the proliferation of misinformation about food and nutrition in venues available to the general public [2–5]. Coupled with the ongoing deluge of new nutrition research findings published in the peer-reviewed literature, the nutrition and broader healthcare communities must have the skills to critically evaluate this literature to discern which information is appropriate for integration into guidelines and practice.

To address this gap, Tufts Clinical and Translational Science Institute (CTSI) created a short, self-paced online continuing education course for nutritionists and dietitians and other interested healthcare professionals titled: Nutrition-related Research: Best Practices for Evaluating and Interpreting Studies (henceforth referred to as Nutrition-related Research). The course is designed to help healthcare professionals develop critical skills to evaluate and integrate nutrition research into practice.

Self-paced online learning is an efficient and effective way to provide continuing education and training for healthcare professionals in a variety of contexts and disciplines and frequently allows those needing continuing education credits to fulfill their requirements [6–9]. The two-module course is based on a series of papers that outline core principles for designing and conducting human nutrition randomized controlled trials (RCTs) [10–14]. The course provides basic information for 1) identifying and critically evaluating peer-reviewed nutrition research, 2) differentiating among sources of evidence, 3) interpreting research results and making decisions about their integration into guidelines and practice, and 4) understanding the structure and importance of RCTs in nutrition research. The course is available at no cost through the Tufts Clinical and Translational Science Institute (CTSI) I LEARN platform(====https://ilearn.tuftsctsi.org/?category=3&keyword=nutrition&cond=advand&search=GO). Nutrition and dietetics professionals (i.e., Registered Dietitians/Registered Dietitian Nutritionists and Dietetic Technicians) can earn 1.5 continuing education credits from the Commission on Dietetics Registration upon successful course completion.

The course became publicly available in April 2022; however, by January 2023, there was minimal course participation, with 23 learners having completed any course content. The Commission on Dietetic Registration reports a community of more than 115,000 registered nutrition and dietetics professionals [15]. Given the large size of the potential learner population, we expected the course to be one of the more popular on the Tufts CTSI I LEARN platform, where top courses typically engage 100 or more learners per year. Without learners’ use of the course, it would be unable to achieve its goal of providing the skills needed to critically evaluate and integrate emerging nutrition literature. To address the issue of lower than desired engagement, we conducted a quality improvement project using Dissemination and Implementation (D&I) science principles to develop and carry out a purposeful course dissemination plan to increase course enrollment. The plan expanded our initial target audience of registered dietitians to include university nutrition program instructors (“instructors”) and their trainees. This article outlines our approach, serving as an illustration of how D&I methods and frameworks can be used to promote the dissemination and implementation of educational activities and interventions.

### Dissemination and Implementation Science

A key challenge in online educational interventions is ensuring they reach and engage the intended audience. While there are many approaches to quality improvement, we selected D&I science principles because they offer a structured, evidence-based framework for promoting the adoption of innovations—such as our Nutrition-related Research course—among diverse audiences. D&I science is particularly well-suited for identifying barriers to engagement in complex systems and developing strategies to ensure that educational content effectively reaches learners [16]. Its systematic methods allow for careful planning, monitoring, and adjusting of dissemination strategies to maximize participation and impact. The proven success of D&I frameworks in facilitating the uptake of evidence-based practices in healthcare settings underscores their relevance for guiding improvements in this educational activity [17–19].

**Figure 1.**
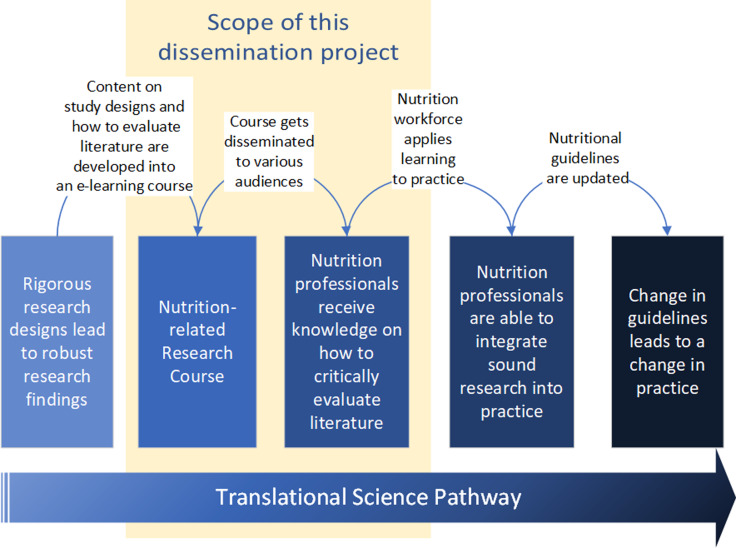
Scope of this dissemination project along the translational science pathway.

D&I science is characterized by its use of models and frameworks to plan, execute, and evaluate dissemination and implementation activities. Process frameworks help organize the planning process, while determinant frameworks facilitate the identification of dissemination barriers and facilitators, and potential dissemination strategies to optimize reach and engagement. Typical D&I plans include strategies to share key information with specific audiences across defined channels. Evaluation measures are essential, allowing the team to assess the effectiveness of the dissemination strategies in achieving overall uptake. This approach demonstrates how D&I principles, combined with quality improvement methods, can guide the dissemination and improvement of educational innovations, ensuring their sustained adoption and impact. Fig. 1 illustrates the scope of this dissemination project along the larger translational science pathway.

## Methods

This quality improvement project aimed to enhance the dissemination of the Nutrition-related Research course by applying D&I science principles. We began by engaging key stakeholders, including potential course implementers and users, to gather their perspectives on course dissemination. These insights informed the development of our dissemination plan, which was structured using D&I frameworks to ensure a systematic approach. We then implemented targeted dissemination strategies based on stakeholder feedback and continuously collected data to track course usage, assess the effectiveness of our dissemination efforts, and inform future course improvements. The project was determined to be nonhuman subjects research by the Tufts Health Sciences Institutional Review Board.

### Dissemination Team

The dissemination team included members with diverse perspectives to ensure a comprehensive approach to course dissemination. The course content developers provided input on potential stakeholders, including possible implementers and adopters who could provide their perspectives on barriers and facilitators to course dissemination and adoption. Tufts CTSI’s Professional Education team members, with expertise in instructional design, developed an engaging learning experience. Experts in D&I science coordinated dissemination plan development and the iterative improvement activities.

### Dissemination and Implementation Models and Frameworks

To promote the dissemination and implementation of the course, we employed a four-phase process adapted from the Value-Added Research Dissemination Framework [20]. This framework provides a structured approach to address potential dissemination barriers and effectively reach key audiences [20]. It views dissemination as a communication process, emphasizing how tailored communication strategies can enhance success. The four phases—planning, translation and packaging, strategic distribution, and follow-through and evaluation—guided our efforts to plan, translate, and distribute the course. We adapted the fourth phase to include continuous cycles of assessment and improvement. Additionally, we used the Consolidated Framework for Implementation Research (CFIR) to systematically identify dissemination barriers and potential dissemination strategies. CFIR’s five domains—intervention characteristics, inner setting, outer setting, characteristics of the individuals, and implementation process—to help us characterize key barriers and facilitators [21].

### Planning Phase

During the planning phase, we engaged stakeholders to identify the target audience for the course, determine the information they need, and understand how they might receive course information. We conducted informal discussions with seven stakeholders selected based on their nutrition research, education, or leadership expertise, and their ability to provide insights into the potential role of the course in graduate and undergraduate nutrition programs, and for nutrition/dietetic continuing education credits. Key audiences for the course were identified as practicing dietitians, nutrition and dietetic undergraduate and graduate students, dietetic interns, and instructors interested in learning or teaching how to evaluate peer-reviewed nutrition research studies. Instructors, representatives from the Accreditation Council for Education in Nutrition and Dietetics (ACEND), and dietetic professional group leaders suggested strategies and messages to reach these audiences. These discussions, framed by CFIR domains, helped us understand the relevance of course characteristics, stakeholders’ needs when implementing or using the course, and external policies that influence nutrition education program standards and requirements for dietetic professional continuing education.

### Translation and Packaging Phase

The translation and packaging phase involved tailoring dissemination, implementation, and communication strategies to address barriers and meet audience needs. Informed by the planning phase, we developed a multipronged dissemination approach to reach practicing dietitians, instructors, and their students. Table 1 illustrates potential barriers, facilitators, and related D&I science strategies. For registered dietitians, the dissemination messages highlighted the availability of continuing professional education credits, presenting the course as a means to enhance their ability to interpret emerging nutrition research findings and become more skilled practitioners. To reach students, we targeted instructors with curricula closely aligned with the course, providing a one-page summary demonstrating how the course met key ACEND learning standards to gain instructor buy-in for adoption.

**Table 1.** CFIR domains and constructs, identified facilitators and barriers, and dissemination strategies

CFIR domain and construct	Facilitators (+) and barriers (−)	Dissemination strategies
Innovation: relative advantage	Few similar courses are available to nutrition programs and practicing dietitians (+)	− Targeted instructors and practicing dietitians interested in a course that fills the gap in evaluating recent nutrition research findings.
Innovation: design	Simple course design and engaging learning platform (+) Course is difficult to locate online (−)	− Course designed in a professional learning management platform. − Monitored user feedback on ease of use. − Dissemination materials contained direct link to course on learning platform.
Outer setting: external policy requirements	Need for continuing professional education credits (+)	− Course designed to meet credentialing requirements. − Continuing education credits, at no cost, incentivized learners. − Marketing materials targeted registered dietitians.
Outer setting: external policy requirements	ACEND learning standards for nutrition education programs (+)	− Course designed to meet ACEND standards. − Instructors received a one-page summary on how the course meets ACEND learning standards for nutrition research education programs
Outer setting: local attitudes	Continuing education on research is not always viewed as relevant to daily work (−)	− Targeted instructors to integrate course in their curricula, addressing low initial interest among practicing dietitians.
Inner setting: compatibility	Curricula already established for nutrition programs (−)	− Free online course is compatible with existing curricula.
Inner setting: incentive system	Course was required in pilot nutrition programs (+)	− Requiring course completion promoted student adoption.
Individuals: innovation implementers and recipients	Innovation implementers (instructors) and students viewed course positively (+)	− Instructors were asked to recommend the course to others to promote further adoption.
Implementation: engaging innovation deliverers	Innovation implementers (instructors) decided how the course would be integrated in curricula (+)	− Instructors were supported in deciding the timing and method of integrating the course into the curricula to align with teaching goals and maximize student engagement.

CFIR = Consolidated Framework for Implementation Research.

### Strategic Distribution Phase

We deployed various strategies across several communication channels to distribute the course. For dietitians, we promoted the course through social media and relevant nutrition-related newsletters. We contacted social media managers of dietetic and nutrition-related program accounts, such as ACEND, the Nutrition and Dietetic Educators and Preceptors Group, and the Friedman School of Nutrition Science and Policy at Tufts University, to maximize our reach. Three instructors, selected through professional networking, agreed to pilot test the Nutrition-related Research course in their programs during the Spring, Summer, and Fall semesters of 2023. We relied on these instructors to promote the course to their students and on educators’ messaging platforms to champion the course to their network of nutritionists and dietitians.

### Assessment and Improvement Phase

**Figure 2.**
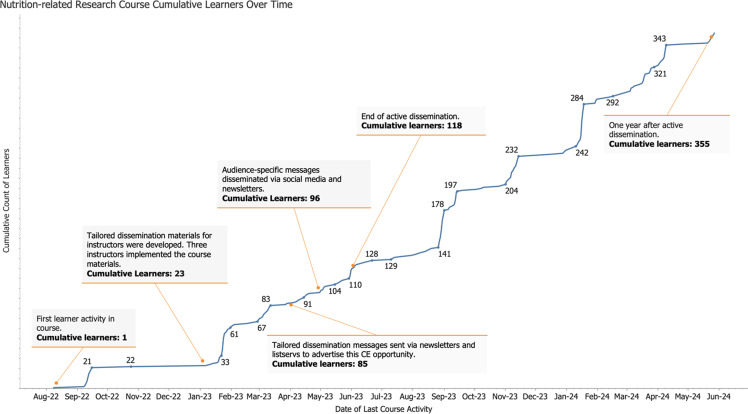
Cumulative number of learners over time with key dissemination activities noted.

During the assessment and improvement phase, we sought to understand the degree to which the course was being used and how dissemination and adoption could be improved. Our team reviewed monthly statistics for course use, plotted these data on a line graph (Fig. 2), and annotated it with specific dissemination activities. We reviewed the data for trends to assess which strategies increased learner engagement, defining active learners as users who interacted with course content beyond merely registering. We continued strategies that led to larger increases in engagement and discontinued those (i.e. social media promotion) that were less effective.

As part of the Professional Education team’s ongoing evaluation and continuous improvement efforts, each course on the Tufts CTSI I LEARN platform includes a short survey upon completion. The surveys assess users’ perceptions of the course, including ease of use and usefulness. Questions are divided into three categories: 1) questions related to the learner’s self-confidence in achieving the course’s learning objectives; 2) questions on the overall course design; and 3) open-ended questions on the most valuable aspects of the course and areas for potential improvement. We analyzed responses from learners who completed the evaluation survey for the Nutrition-related Research course between January 1 and May 31, 2023, to align with the period of active dissemination activities.

Finally, our team conducted informal discussions via Zoom with the instructors who used the course in their undergraduate and graduate nutrition and dietetic internship programs to ascertain their perceptions of the course’s alignment with their curricula and its impact on students’ learning outcomes and engagement.

## Results

At the start of our dissemination activities in January 2023, the course attracted 23 learners. Fig. 2 shows key dissemination activities alongside the cumulative number of learners over time. Direct outreach to nutrition research instructors led to a large initial increase in the number of learners early in 2023. By the end of our active dissemination efforts in May 2023, the cumulative learner count had increased to 118, with 95 of those learners completing the course content between January and May 2023.

The line chart in Fig. 2 highlights several sharp gains in participation that occurred at distinct points during the reporting period. These gains began in approximately late January through early February 2023, shortly after the first dissemination activity was implemented, coinciding with the start of academic semesters and other critical times during the school year. The course was integrated into the curricula of three nutrition programs, where instructors tailored use to fit their teaching styles and program needs. For undergraduate students, the course was offered as a standalone assignment, encouraging individual engagement. Graduate students were required to complete the course as a prerequisite for additional in-class assignments, creating a more structured use of the material. For dietetic interns, the course was incorporated into an in-class teaching session, prior to a specific course assignment, reflecting a more interactive approach.

This variability in implementation likely contributed to the timing and magnitude of the observed gains. Although we lack precise data on when specific groups completed the course, these patterns suggest that coordinated efforts by instructors during semester transitions played a key role in driving learner engagement.

Additionally, the course was available to dietitians seeking continuing education credit during this same time period, which may have contributed to the observed gains in late spring and early summer 2023. While we cannot differentiate between student and registered dietitian usage, the sustained increase in cumulative learners suggests that dissemination efforts reached both audiences effectively.

Importantly, the increase in cumulative course completion continued over the reporting period, with sharp increases in fall 2023 and early 2024 in alignment with the cadence of the academic year, reflecting sustained and potentially expanded adoption. These findings underscore the impact of integrating dissemination strategies into academic programs and professional networks, illustrating how tailored approaches can promote widespread engagement with educational resources.

Our analysis of the learner evaluation surveys indicated that the course was positively received. Of the 95 learners who completed content between January 2023 and May 2023, 93% (88/95) also completed the evaluation survey. Among these, the majority reported high confidence in achieving the course learning objectives, with average confidence ratings ranging from 6.62 to 6.84 on an 8-point scale (see Table 2). In addition, most learners agreed or strongly agreed that the course was well organized, relevant, and stimulated new ideas.

**Table 2.** Learner responses to course evaluation survey, January 1 – May 31, 2023

Learners’ self-confidence in achieving learning objectives (*N* = 88)*				
Learning Objectives	Cannot do	Moderately certain can do	Certain can do	
Define the major types of peer-reviewed publications in nutrition and their relative strengths.	1%	14%	85%	
Describe guidelines for interpreting and applying research results to practice.	3%	13%	84%	
Differentiate among commonly used nutrition interventions.	2%	14%	84%	
Differentiate among the types of study designs used in randomized controlled trials.	3%	16%	81%	
Evaluate clinical nutrition studies that inform guidelines, policy, and practice decisions.	3%	13%	84%	
Learners’ subjective ratings of course content and format**				
	Strongly disagree to Disagree	Neutral	Agree	Strongly Agree
The course was well organized. (*N* = 88)		7%	28%	65%
The material covered was relevant to my research, practice, or work. (*N* = 87)		7%	22%	71%
This course stimulated new ideas for my own research or practice. (*N* = 88)	5%	16%	36%	43%
Overall, I am satisfied with this course. (*N* = 88)		8%	38%	55%
I would recommend this course to others. (*N* = 87)	1%	9%	32%	57%
The Tufts CTSI I LEARN platform was easy to use. (*N* = 87)	1%	6%	33%	60%

* Learners rated self-confidence on an 8-point scale. Ratings of 1-3 = Cannot do, ratings of 4-5 = Moderately certain can do, and ratings of 6-8 = Certain can do.

** Learners rated the course on a 5-point scale: “Strongly disagree,” “Disagree,” “Neutral,” “Agree,” and “Strongly agree.” In this table, strongly disagree and disagree were combined due to the small number of responses.

The responses to the open-ended questions were analyzed and grouped by theme. Learners found that the relevance of the material, its applicability to real-world nutrition practice, and their continued studies and interest in research were the most valuable aspects of the course. Many learners commented on the helpfulness of the diagrams and graphics, particularly with respect to the module on RCTs. The most common suggestion for improving the course was to add audio narration to accompany the text and visuals, followed by requests to include additional examples and a review section before the final test.

The results of the semi-structured interviews revealed that all three of the degree program instructors indicated the course to be a valuable addition to their curricula, noting that it either introduced new content or reinforced existing program material. They also highlighted that the course content was well-designed and aligned with their program goals, enhancing its utility for both students and instructors.

### Conclusion and Future Directions

Nutritionists, registered dietitians, dietetic interns, trainees, and health sciences students need to know how to evaluate peer-reviewed publications for scientific rigor and report to fully understand their contribution to the evidence base. While the content of the nutrition-related research course was explicitly designed to meet this need and satisfy continuing education competencies for nutrition and dietetic professionals, offering the course for free online was insufficient to attract an engaged group of learners. Additional communication and dissemination strategies were necessary to raise awareness of its existence and potential benefits. By using D&I science principles during a brief active dissemination period, we successfully increased course enrollment and sustained adoption for over 12 months.

Conversations with stakeholders helped us tailor dissemination strategies and messaging and led our team to use a multipronged dissemination approach to reach registered dietitians, dietetic program instructors, and current dietetic students. Tracking key engagement activities against enrollment numbers allowed us to iteratively assess and refine our dissemination strategies, exemplifying a quality improvement approach focused on identifying and scaling the most effective interventions to achieve the largest increases in registered learners. We plan to track course use over the next year to determine if the current level of use is sustained, further supporting our dissemination strategy. Given the general interest in food and nutrition information and prevalence of nutrition misinformation, other healthcare professionals may find the information valuable.

Based on the learner feedback and instructor interviews, we plan to rely more on nutrition instructors to embed the Nutrition-related Research course within their existing nutrition degree program curricula and encourage them to continue requiring the course for their students. We will shift our focus from social media dissemination to direct outreach to a network of instructors in the field of nutrition. Additionally, student feedback suggests the course would benefit from audio narration, a feature we plan to incorporate in the next iteration.

Disseminating educational tools to promote skills that enable uptake of research findings is challenging. Prior to our dissemination efforts, there was a limited uptake of the Nutrition-related Research course. By the end of May 2024, 1 year after active dissemination activities concluded, the course had attracted a cumulative enrollment of 468, with 355 learners having completed the course content for an overall retention rate of 76%. This is particularly notable because similar types of free, open-enrollment online education typically have very high dropout rates, sometimes with only 10–30% of enrollees completing a course [22]. The Nutrition-related Research course became the top performer on the Tufts CTSI I LEARN platform in 2023, with 215 learners in that calendar year, compared to an average of 9.9 learners per course across the platform in 2022 [23].

Importantly, integrating the course into existing nutrition educational programs, where the instructors embed it directly into their curricula, offers several advantages. Potential drivers of quality in educational interventions include alignment with learners’ professional needs, ease of integration into existing structures, and adaptability to diverse educational contexts. Embedding the course into curricula not only raises immediate awareness but also ensures sustained engagement over time, as the course becomes a regular part of didactic instruction. Unlike one-time communication efforts, such as newsletters or social media posts, embedding the course within established programs creates a self-sustaining cycle of adoption, with each new cohort of students being introduced to the material. By leveraging the ongoing nature of academic courses, this strategy ensures that the Nutrition-related Research course remains relevant and accessible across multiple semesters, ultimately reaching a larger and more consistent audience.

Leveraging D&I strategies to boost engagement in this course has also had positive downstream effects on our Professional Education program. Building on this success, we have created a dissemination plan template that is now mandatory for all new and revised courses. This template walks course developers through seven common dissemination plan elements, such as communication channels, dissemination materials, and long-term engagement, with a series of more specific questions tailored to the different course modalities supported by the Professional Education team (live, blended synchronous/asynchronous, and self-paced online). We use the strategies employed in the dissemination of the Nutrition-related Research course as examples and inspiration for other course dissemination plans. Additionally, we plan to apply these methods to other courses, including the “Community Participation in Research Course,” which orients community members to translational research.

Two main limitations should be considered in this quality improvement project. We held targeted informal discussions with a small, carefully selected group of stakeholders to gather in-depth insights into potential barriers and facilitators for course dissemination and adoption. This small sample size may limit generalizability but provides crucial context-specific information. The project aimed to increase engagement with our online course through targeted dissemination strategies. While we observed increased course usage, we cannot infer that one strategy was superior to another since they were deployed in parallel. Despite these limitations, the insights gained from this project provide valuable guidance for future quality improvement initiatives to enhance the adoption of online education.

This work advances the application of D&I frameworks to educational innovations. Future research could explore how different D&I science strategies—such as implementation facilitation or peer-led dissemination—impact the scalability of educational tools in Clinical and Translational Science. For example, hybrid trials could be designed to compare dissemination approaches for education-focused interventions across sites, generating empirical data to guide their implementation. Ultimately, the integration of quality improvement, D&I science, and Clinical and Translational Science approaches offers a path forward for developing robust, scalable educational resources that empower professionals to engage with and apply research findings in their practice.

## References

[1] Abu-Odah H , Said NB , Nair SC , et al. Identifying barriers and facilitators of translating research evidence into clinical practice: a systematic review of reviews. Health Soc Care Community. 2022;30(6):e3265–e3276. doi: 10.1111/hsc.13898. 35775332

[2] Handu D , Moloney L , Rozga M. Combating diet misinformation through navigating the complexity of prescribed versus actual dietary intake in nutrition research. J Acad Nutr Diet. 2023;123(10):A16. doi: 10.1016/j.jand.2023.08.032.

[3] Lopez T , Boutros B , Brodeur T , VanderKaay D , Browning-Keen V. The impact of social media on dietary behaviors of college students: a qualitative approach. J Acad Nutr Diet. 2022;122(9):A30. doi: 10.1016/j.jand.2022.06.112.

[4] de Jesus JM , Stoody EE , DeSilva DM , et al. Addressing misinformation about the dietary guidelines for Americans. Am J Clin Nutr. 2024;11(19):1101–1110. doi: 10.1016/j.ajcnut.2024.02.034. 38522617

[5] Commission on Dietetic Registration. 2020-2025 Essential practice competencies. Accessed August 8, 2024. https://admin.cdrnet.org/vault/2459/web/New_CDR_Competencies_2021.pdf.

[6] Yoo J , De Gagne JC , Kim HJ , Oh J. Development and evaluation of a web-based acute pain management education program for Korean registered nurses: a randomized controlled trial. Nurse Educ Pract. 2019;38:7–13. doi: 10.1016/j.nepr.2019.05.013. 31170627

[7] Shafto K , Shah A , Smith J , et al. Impact of an online nutrition course to address a gap in medical education: a feasibility study. PRiMER. 2020;4:5. doi: 10.22454/PRiMER.2020.368659. 32537605 PMC7279113

[8] Hurtado SL , Simon-Arndt CM , Belding JN , Sanchez SS , Spevak C , Osik A. Evaluation of two educational modalities for the clinical practice guideline for opioid therapy for chronic pain for US military physicians. J Contin Educ Health Prof. 2023;43(4):241–246. doi: 10.1097/ceh.0000000000000476. 36728977 PMC10664779

[9] Stark CM , Garner CD , Garg A , Bégin F. Building capacity of health professionals in low- and middle-income countries through online continuing professional development in nutrition. J Contin Educ Health Prof. 2021;41(1):63–69. doi: 10.1097/ceh.0000000000000334. 33560042 PMC7919702

[10] Weaver CM , Lichtenstein AH , Kris-Etherton PM. Perspective: guidelines needed for the conduct of human nutrition randomized controlled trials. Adv Nutr. 2021;12(1):1–3. doi: 10.1093/advances/nmaa083. 33200180 PMC7850095

[11] Lichtenstein AH , Petersen K , Barger K , et al. Perspective: design and conduct of human nutrition randomized controlled trials. Adv Nutr. 2021;12(1):4–20. doi: 10.1093/advances/nmaa109. 33200182 PMC7849995

[12] Weaver CM , Fukagawa NK , Liska D , et al. Perspective: US documentation and regulation of human nutrition randomized controlled trials. Adv Nutr. 2021;12(1):21–45. doi: 10.1093/advances/nmaa118. 33200185 PMC7850145

[13] Maki KC , Miller JW , McCabe GP , Raman G , Kris-Etherton PM. Perspective: laboratory considerations and clinical data management for human nutrition randomized controlled trials: guidance for ensuring quality and integrity. Adv Nutr. 2021;12(1):46–58. doi: 10.1093/advances/nmaa088. 33200184 PMC7849975

[14] Petersen KS , Kris-Etherton PM , McCabe GP , Raman G , Miller JW , Maki KC. Perspective: planning and conducting statistical analyses for human nutrition randomized controlled trials: ensuring data quality and integrity. Adv Nutr. 2021;12(5):1610–1624. doi: 10.1093/advances/nmab045. 33957665 PMC8483948

[15] Commission on Dietetic Registration. Registry statistics. https://www.cdrnet.org/registry-statistics, Accessed January 10, 2025.

[16] Shelton RC , Lee M , Brotzman LE , Wolfenden L , Nathan N , Wainberg ML. What is dissemination and implementation science?: an introduction and opportunities to advance behavioral medicine and public health globally. Int J Behav Med. 2020;27(1):3–20. doi: 10.1007/s12529-020-09848-x. 32060805

[17] Rositch AF , Unger-Saldaña K , DeBoer RJ , Ng‘ang’a A , Weiner BJ. The role of dissemination and implementation science in global breast cancer control programs: frameworks, methods, and examples. Cancer. 2020;126(S10):2394–2404. doi: 10.1002/cncr.32877. 32348574

[18] Lambert-Kerzner AC , Aasen DM , Overbey DM , et al. Use of the consolidated framework for implementation research to guide dissemination and implementation of new technologies in surgery. J Thorac Dis. 2019;11(Suppl 4):S487–s499. doi: 10.21037/jtd.2019.01.29. 31032067 PMC6465432

[19] Curtis K , Kennedy B , Considine J , et al. Successful and sustained implementation of a behaviour-change informed strategy for emergency nurses: a multicentre implementation evaluation. Implement Sci. 2024;19(1):54. doi: 10.1186/s13012-024-01383-7. 39075496 PMC11285323

[20] Macoubrie J , Harrison C. The value-added research dissemination framework. 2013, https://www.acf.hhs.gov/sites/default/files/documents/opre/valueadded.pdf.

[21] Damschroder LJ , Aron DC , Keith RE , Kirsh SR , Alexander JA , Lowery JC. Fostering implementation of health services research findings into practice: a consolidated framework for advancing implementation science. Implement Sci. 2009;4(1):1–15. doi: 10.1186/1748-5908-4-50. 19664226 PMC2736161

[22] Reich J , Ruipérez-Valiente JA. The MOOC pivot. Science. 2019;363(6423):130–131. doi: 10.1126/science.aav7958. 30630920

[23] Markman KM. Educating the clinical and translational research workforce online: a case study of tufts CTSI I LEARN. J Clin Transl Sci. 2023;7(s1):25–26. doi: 10.1017/cts.2023.174.

